# Calculus in Sublingual Gland

**Published:** 1897-01

**Authors:** S. L. Goldsmith


### Domestic Correspondence.

### CALCULUS IN SUBLINGUAL GLAND.

TO THE EDITOR:

SIR,—The paper by Dr. Fütterer on salivary calculi, in the October number of your JOURNAL, brings to my mind a case which came to me for treatment some time ago. Miss H., aged about twenty-one years, presented herself suffering with pain in a right lower incisor: as the tooth did not respond to applications of chloride of methyl, it was opened, and the tooth found to be in a dying but not putrescent condition. After removing the pulp, the lower third of which was in a comparatively normal state, a dressing of cassia oil was inserted, and the patient dismissed.

The next day the patient presented herself with face somewhat swollen and no diminution of pain. As to my mind the pain could no longer be from the tooth, I made a careful examination of the mouth and found an abscess of the right sublingual gland "pointing" towards the median line. Dr. Felix Cohn, who saw the case in consultation, expressed the opinion that the abscess was a result of infection from the tooth. After incising the abscess and evacuating the pus the gland was probed, but nothing discovered.

The wound was then packed with gauze and the patient ordered to return that evening. On removing the gauze a hard object was seen which proved to be a calculus the size of a cherry pit. The next morning another calculus somewhat smaller in size was removed.

Now the question which arises is, Was the fact that the gland abscess and the pulpitis took place about the same time, as in my opinion, merely a coincidence, or did the suppuration ensue in consequence of the invasion of micrococci from the tooth?

Dr. Cohn held the opinion that while of course the calculi must have been present for a long time, there would have been no pus-formation until the infection took place.

S. L. Goldsmith.

